# Query3d: a new method for high-throughput analysis of functional residues in protein structures

**DOI:** 10.1186/1471-2105-6-S4-S5

**Published:** 2005-12-01

**Authors:** Gabriele Ausiello, Allegra Via, Manuela Helmer-Citterich

**Affiliations:** 1 Centre for Molecular Bioinformatics, Department of Biology, University of Rome Tor Vergata, Via della Ricerca Scientifica, 00133 Rome, Italy

## Abstract

**Background:**

The identification of local similarities between two protein structures can provide clues of a common function. Many different methods exist for searching for similar subsets of residues in proteins of known structure. However, the lack of functional and structural information on single residues, together with the low level of integration of this information in comparison methods, is a limitation that prevents these methods from being fully exploited in high-throughput analyses.

**Results:**

Here we describe Query3d, a program that is both a structural DBMS (Database Management System) and a local comparison method. The method conserves a copy of all the residues of the Protein Data Bank annotated with a variety of functional and structural information. New annotations can be easily added from a variety of methods and known databases. The algorithm makes it possible to create complex queries based on the residues' function and then to compare only subsets of the selected residues. Functional information is also essential to speed up the comparison and the analysis of the results.

**Conclusion:**

With Query3d, users can easily obtain statistics on how many and which residues share certain properties in all proteins of known structure. At the same time, the method also finds their structural neighbours in the whole PDB. Programs and data can be accessed through the PdbFun web interface.

## Background

A lot of information on the relationship between structure and function lies hidden in the high number of known protein structures. Protein local structure comparison methods are powerful instruments in helping elucidate the mechanisms that connect protein structural features to the protein's function. Comparison methods can highlight correlations between spatial positioning of single aminoacids and their interactions with the surrounding environment.

In the last ten years many new and highly effective comparison methods have been developed (for a review see [1]). Since speed is one of the most treated and fundamental features, some of these methods are now able to search accurately a structural motif in the whole set of known structures in a very short time.

However the ability to provide and embed biological information in the comparison algorithm should be considered even more important than speed. To accomplish this, a high degree of integration of databases and functional annotation programs is needed. Many comparison methods do not treat integration aspects and, by concentrating their efforts on the comparison algorithm, consider aminoacids independently of their biological context. Often protein residues are described as set of points associated with physico-chemical characteristics, with no additional information on their real or supposed functions.

The structural biologist who uses local comparison methods to find similarities between a specific protein of interest and a database of structures, needs to access residues biological function and properties in two different phases: when choosing the structural pattern of the query protein and when analyzing the comparison results in search of a biological rationale for the structural similarities.

The biological information shared by comparison methods is so poor that users need to do a lot of manual browsing among different databases both before and after the structural comparison. If the comparison is not between two single proteins or motifs but between a structure and a set of structures, or between a set of motifs and a database of structures, the manual work needed for the analysis of the results increases rapidly and becomes unaffordable in the case of high-throughput analyses.

Some of the developed methods in the pre-run phase provide the user a number of selected sets of structural motifs to search with. The most frequent case is the one where the user is given a single list of structural motifs automatically extracted from a single database. PDBSITESCAN [2] gives a list of 8000 structural motifs, some of these automatically extracted from the SITE field of the PDB [3] and others obtained by analyzing protein-protein contacts. TESS [4] uses the PROCAT database of enzyme active sites as a list of structural motifs. CAVBASE [5] is based on the RELIBASE database [6] of protein-ligand interactions. The comparison method developed by Kinoshita et al. [7] uses the EF-SITE database [8] of automatically predicted functional sites.

WEBFEATURE [9] runs on a set of automatically predicted binding sites. PINTS [10] makes it possible to search for structural motifs from four different lists: SCOP families, interactions with PDB hetero-atoms, PDB SITE fields and exposed residues. Apart from the last two described methods, which are accessible through a web interface, comparison methods are not generally developed for specific applications and are not easily available for public use.

None of the cited methods makes it possible to combine or integrate information coming from different lists of motifs or databases by allowing the users to search for sets of residues characterized by properties of different types (i.e.: the solvent-exposed residues of a PROSITE motif).

A comparison method along this lines is ASSAM [11]. ASSAM gives the user many features on structural residues. Such features can be used for the search: information from the SITE field, secondary structure from DSSP [12], disulphide bridge from SSBOND field, and solvent accessibility calculated on biological quaternary structure. Moreover, ASSAM allows the user to partially combine different features in the same structural search. For example, secondary structure of the matching residues can be specified.

Here we describe Query3d, a new method that integrates many existing databases and programs for 3D functional annotation together with a fast structural comparison algorithm. Nine data sources have been interconnected ranging from solvent exposure to ligand binding ability, location in a protein cavity, secondary structure, functional pattern, protein domain and catalytic activity. All this functional information is bound to the single residue and not to the structure as a whole, allowing the user to perform detailed queries on the features of single residue sets. All the structural and functional data are stored locally and managed by a fast and powerful database management system which is also able to perform fast and high-throughput local structural comparisons.

## Results

### Overview

Query3d is both a database management system (DBMS) oriented to protein structural analysis and a structural comparison algorithm. These two features can be used individually or combined, giving rise to three types of analysis, as described below.

The first option is the use of Query3d as a local structural comparison program. Regions of local similarity can be searched between any pair of protein structures, between a protein chain and the whole PDB or also between any two arbitrary and chosen subsets of aminoacids in a structure.

The second possibility consists in the use of Query3d as a DBMS devoted to the functional analysis of protein structures. The program provides access to a rich database of functional and structural information on all PDB residues. Users can create arbitrary complex queries on all known structures. For instance, users can ask about the number and identity of the residues sharing a chosen set of properties. A typical query subset can consist of all residues that are able to bind ATP or ADP, are not hydrophobic and belong to a loop. The program returns the total number of such residues per chain in the whole PDB (useful for structural statistical studies) and selects these residues for further analysis.

However, the most interesting application of our method is obtained from the combination of DBMS and the comparison algorithm. By using these two features at the same time, users can create automated and customized selections of functional residues to be searched for structural similarity with the whole PDB or with other residue selections. For instance, the previously described binding sites can be compared with all residues lying in the major cavities on the surface of a set of catalytic domains.

### Dataset used

Structural and biological data for comparison and functional querying was derived using the PdbScan package (manuscript in prep.). PdbScan is a set of programs created to build a common interface and access method to the PDB structure database and the major existing databases and methods of proteins functional/structural annotations. PdbScan output is a residue-oriented relational database where all protein residues with their main characteristics extracted from the PDB are stored together with other information mapped from other data sources. In order to generate these data, PdbScan runs locally a variety of annotation methods or imports functional information on protein structure from different existing databases. Each different source of data information is called a feature. Examples of residue features present in PdbScan are: secondary structure, solvent accessibility, conservation, interaction with a ligand and position in a protein domain or in an enzyme active site. For each feature present in the database, a single value is assigned to each PDB residue. For example, a residue can bind ATP, can be solvent-accessible, can be present in an SH3 structural domain and share a certain conservation value in a multiple alignment of homologous proteins.

### Queries

The program can run two types of user queries: simple or complex ones.

Simple queries involve only a single feature. By using this type of query, users can select all residues sharing a single common annotation or any number of annotations belonging to the same feature. For example, with the 'binding sites' feature, users can select all residues interacting with ATP, but also all residues interacting with ATP or ADP or Phosphate. The complete selection of all the annotations of a feature is also possible, e.g., all residues involved in the binding of any ligand.

Complex queries can be created by combining pairs of simple queries generated with selections in different features. Combinations are created using Boolean operators AND, OR and NOT. We propose an example of a complex query that combines, with an AND operation, a simple query on the 'binding sites' feature with a simple query on the 'secondary structures' feature. This query could select all residues in the PDB that are located in an alpha helix and are able to bind ATP or ADP. Complex queries can also combine other complex queries and not only simple ones.

After each selection (simple or complex query) the DBMS can return three different levels of information: i) the total number of PDB residues selected by the query and the total number of PDB chains sharing at least one selected residue; ii) the complete list of chains with at least one selected residue together with the total number of residues selected in each chain; iii) the complete list of selected residues, together with the complete list of annotations of each residue, in each selected protein chain.

### Query example

Selecting 'ATP' and 'ADP' in the 'binding sites' feature we obtain a list of all the 8998 residues distributed among 840 chains of the PDB that are able to bind ATP or ADP.

Selecting 'hydrogen bonded turn' in the '2D structures' feature we obtain a second list of all the 1400109 residues in 49278 chains that are in a 'loop'.

By operating an intersection using the 'AND' operator between the two list of residues we obtain a new list of 1164 residues in 570 chains that are in a loop and are also involved in ATP or ADP binding.

### Comparison algorithm

The structure comparison method in Query3d is designed to find the largest subset of matching aminoacids between two complete protein chains or between two sets of selected residues. The program can search for structural local similarity between selected residues of any pair of user queries. The matching process is completely sequence independent so local similarity has to be intended between residues that are neighbours in space. The output of the program is, for each pair of compared chains, a list of the residues found to be similar. The detailed description of the comparison algorithms is given in 'Methods'.

The method was found to function correctly in previous authors' works. The method was applied in different test cases and proved capable of finding significant local structural similarities, even in the absence of protein sequence or protein fold similarities. More specifically, the algorithm proved capable of recognizing five different known difficult cases of local structural similarity and has been extensively used in a structural genomics function prediction experiment [13]. In [13], the method has also been applied to the search of similarities between a complete collection of annotated surface clefts extracted from the PDB and the whole database of annotated and non annotated surface clefts. Only 2% of the high-scoring matches can be considered as possible false positive hits or not yet identified true positives.

### Availability

Query3d is open source. It can be accessed through the web or, if special conditions of use are required, Query3d can be installed locally.

A server running the program can be accessed through a web interface. The web interface is called pdbFun [14] and is available at the address . Through the pdbFun interface, all major features of Query3d are available. Help and tutorials in the website facilitate use. Selections of residues can be created and listed in tables. Users can combine selections and manually refine them by adding and removing single residues. Structural comparisons can be launched between selections while structural matches can be visualized instantly. Moreover in pdbFun a java viewer of protein structures is provided to help the user in selecting residues and analyzing structural comparison results.

There are two cases where local installation of Query3D becomes necessary: the need to perform long and computationally intensive structural comparisons or to calculate a large number of selections or comparisons. The public server cannot guarantee all the CPU time needed for an all-versus-all comparison. Or, if many different selections of residues have to be generated and compared, running a batch job on a personal computer is the fastest and most effective thing to do.

The software is available for UNIX/linux platforms. Communication with the server program is carried out through text files and the PostgreSQL database.

## Conclusion

We have developed Query3d. By using this program, the structural biologist can easily select a set of interesting residues according to their biological or structural properties in the whole PDB. The selected residues can be analyzed, counted and manually modified. When the user is satisfied with the selections, structural comparisons can be launched.

Query3d is a new and flexible methodology dedicated to the study and analysis of protein structures. Given the amount of functional information associated to each residue, the method can give an answer to an extremely high number of possible questions of biological relevance. The number of possible combination of queries is so high that it is difficult to envisage all the possible applications of the method.

Future directions include a higher degree of flexibility in the type of possible residue searches. For example, we are going to introduce pattern matching on arbitrary residue features. Possible patterns could be defined in protein-sequence or in a volume of space. These could use not only residue type but also residue features, such as secondary structure or solvent accessibility. The final goal is to transform Query3d in an instrument to search with simple operations in the space of known protein structures, for arbitrarily chosen functional and structural conformation of residues.

## Methods

### Available features

Query3d loads annotated residues data together with residue coordinates and other structural information. An important characteristic of the program is its being independent of the type of data stored. So different versions of PdbScan data can exist, containing different functional information, or even customized features. If a version of PdbScan is used where ten features are implemented, each residue can share a maximum of ten annotations.

Features available are those currently generated by the PdbScan package. They embed nine data sources: solvent exposure as given by the naccess program [15], surface clefts calculated with the SURFNET program [16], protein domains from SMART database [17], secondary structure derived by using the dssp program, PROSITE patterns [18], binding hetero atoms from the PDB, active sites from the CatRes database [19], residue conservation from HSSP [20] and protein fold from CATH [21].

### Protein structure representation

Each protein chain in the PDB is stored locally and described as a set of non-connected, and therefore sequence independent, residues. Each residue is characterized by a set of attributes, such as type of residue, list of neighbour residues, position in space and a list of functional/structural information. Two residues are considered neighbours if the distance between their C alpha atoms is less than 7.5 Angstroms. Two points describe the three-dimensional position of each residue: the first one corresponds to the C alpha atom, while the second is calculated as the geometric average of all the residue side chain atoms. This second set of coordinates gives information on the direction in space in which the side chains are pointing. The last type of information is a complete list of functional and structural properties of the aminoacid in this structure. This information comes from PdbScan (see previous paragraph) and is used by Query3D to permit the users to select the residues that have to be counted or considered for a structural comparison (see next paragraph).

### Structural matching

During the comparison, the program tries to match the maximum number of residues between two protein chains. Two sets of residues are considered similar if they fulfil three criteria: neighbourhood, structural similarity and biochemical similarity.

The first criterion demands that all residues present in the set of matching aminoacids are neighbours of at least one of the other residues in the set. This guarantees that all matched aminoacids are neighbours in space, saving a lot of comparison time. The biological motivation for this constraint is that local comparison algorithms are always used to find similarities between active sites, binding sites and other localized areas in protein structures. In these regions, two areas of distant residues are not expected to be conserved.

The second matching criterion concerns structural similarity. This demands that all sets of matched residues have a root mean square deviation (r.m.s.d.) lower than a certain threshold. The lower the threshold, the faster the program, since a higher number of matches is excluded in the early stages of comparison without the need to explore them further (see next paragraph). However, using a too low r.m.s.d. threshold increases the probability of missing evolutionarily distant similarities (not so well structurally conserved). The present threshold is 0.7 Angstrom and represents a good compromise between speed and accuracy. The r.m.s.d of the match is calculated by using all the matched residue points, both the C alpha and the side chain points. The inclusion of a side chain point in the calculation of the global match r.m.s.d. ensures that also side chains' direction needs to be conserved between two sets of matching residues.

The last criterion is based on the biochemical similarity of residue types. To evaluate this type of similarity, we use a substitution matrix. The default matrix is the Dayhoff one [22]. According to this matrix, if two aminoacids of different type are matched in space, a substitution value is calculated. Two aminoacids cannot be matched if their similarity according to this matrix is lower than 0.3. Moreover, the average substitution value of all the aminoacids belonging to the structural match must be lower than 1.2. Obviously if the user needs a different matrix, also those threshold values need to be changed accordingly.

### Comparison algorithm

Given two protein structures, Query3D is guaranteed to find the two largest sets of matching residues that fulfil the matching criteria described in the previous paragraph. In order to do so, an exhaustive depth-first search is performed exploring all the possible combinations of aminoacids belonging to the two different proteins.

The algorithm starts by creating all the possible length 1 matches. These are composed of a single aminoacid from the probe structure matched to a single aminoacid belonging to the target structure. For example, if the probe and target structures are composed of n and m aminoacids, respectively, n × m length 1 matches can be generated. All these matches are evaluated using the matching criteria and, in case, discarded. Only those matches that are considered valid are extended.

Match extension consists of the generation of new possible length 2 matches starting from each valid length 1 match. All possible pairs of neighbour aminoacids are added to the first two. For example, let the first aminoacids in the probe structure have i neighbours and the corresponding matched aminoacid in the target structure have j neighbours. The algorithm generates i × j new matches of length 2. Again all these new matches are evaluated and, if possible, iteratively further extended to length 3 matches. The process of validation and extension is repeated until no more valid matches can be generated. At this point, the algorithm stops and saves the longest match found. By doing so, the algorithm guarantees that all possible combinations of subsets valid for a structural match have been explored.

Note that in the match extension phase only residues selected by the user are considered among all the available neighbours. This simple fact ensures that structural matches can only include aminoacids that have been chosen by user queries. In order to save time when comparing very similar protein chains, the algorithm is stopped when a match reaches a certain number of residues. We noticed that a match size limit of 10 residues is enough to prevent the program from spending too much time trying all possible combinations of good matches among globally similar structures.

### Implementation

One important feature of Query3d is its speed both in running queries and in comparing structures.

All queries can be run in a few tenth of a second. PDB actual size exceeds 12 million aminoacids, and this performance would not be possible using common databases. Query3d relies on a fast queries algorithm in C that avoids all disk accesses. All the essential information necessary for performing queries and structural comparison is stored in a compressed format in less than 1 GB of RAM. We reckon that with this compression level, foreseeable size increase in PDB in the near future will remain compatible with RAM sizes available for simple desktop PCs.

Protein structure superposition has been optimized. We managed to keep structural comparison time very low. The time needed to compare a protein structure composed of 200 residues with a medium size protein chain in the PDB is only 60 milliseconds on a common 3 GHz pentium4 processor. The search for a local structural similarity between a 200-residue protein and a non-redundant PDB database, at 30% identity, composed of about 4000 structures, can be completed in less than 4 minutes. To obtain these results we developed an optimized C function implementing the quaternion method for calculating the r.m.s.d. between two set of points [23]. We differentiate from the original method, because we do not need the eigenvector of the 4 × 4 quaternions matrix (containing the superposing rotation) but just the lowest eigenvalue that represents the s.r.s. (sum of residual squared) of the best superposition. We can find it merely by solving a quartic equation instead of using the longer algorithms used to find eigenvectors.

## Authors' contributions

AV participated in the design of the work. MHC participated in the design of the work and helped to draft the manuscript. All authors read and approved the final manuscript.
